# The Implementation Experience of COVID-19 Rapid Antigen Testing in a Large-Scale Construction Project in Victoria, Australia

**DOI:** 10.1007/s43477-023-00085-4

**Published:** 2023-05-30

**Authors:** Teralynn Ludwick, Nicola Stephanie Creagh, Jane L. Goller, Claire Elizabeth Nightingale, Angeline Samantha Ferdinand

**Affiliations:** 1 Centre for Epidemiology and Biostatistics, Melbourne School of Population and Global Health, University of Melbourne, Melbourne, Australia; 2 Centre for Health Policy, Melbourne School of Population and Global Health, University of Melbourne, Melbourne, Australia; 3 Microbiological Diagnostic Unit Public Health Laboratory, Department of Microbiology and Immunology, The University of Melbourne at The Peter Doherty Institute for Infection and Immunity, Melbourne, Australia

**Keywords:** Coronavirus, Implementation science, Evaluation, Rapid antigen testing, RE-AIM

## Abstract

The coronavirus (COVID-19) pandemic has caused major disruptions to industries and workplaces. Rapid Antigen Tests (RATs) for COVID-19, which allow individuals to self-administer tests and receive timely results without laboratory testing, provide the opportunity for surveillance testing of asymptomatic individuals in non-medical settings. However, the literature offers few lessons regarding how to create enabling conditions for effective and sustainable implementation in a workplace setting. Guided by the RE-AIM framework, we assessed factors associated with the adoption, implementation, and maintenance of mandatory RAT in a large-scale construction project in Victoria, Australia. We used a mixed methods approach involving site observation, worker surveys (*n* = 30), and interviews with 51 site workers and managers to understand the implementation experience. Factors which facilitated adoption included easy, non-invasive testing procedure; sense of workplace safety; and strong backing by management and acceptance by workers that RATs helped limit COVID-19-related lost days of work. Gaps in knowledge and adherence to testing protocols, logistical challenges (test kit supply, observation of test results), and low appetite for long-term, mandatory testing emerged as challenges for effective implementation and sustainability. As RAT becomes normalized in a range of workplace settings, strategies will be required to support the sustainability of implementation, including longer-term acceptability of surveillance testing and adherence to testing protocols.

**Supplementary Information:**

The online version contains supplementary material available at 10.1007/s43477-023-00085-4.

## Introduction

Since early 2020, the coronavirus (COVID-19) pandemic has caused major disruptions to industries and workplaces, with costs to the economy and individual incomes (Ingram et al., 2021). Workplaces have been a major source of COVID-19 transmission and subsequently been subject to closures, limits on number of employees on site, and other operating restrictions (Lei et al., 2020). Restrictions on industry were particularly tight in Australia, where government lockdowns in the state of Victoria prohibited all but a few essential services and their employees to work onsite, with all other individuals required to work or stay at home (Stobart and Duckett, 2021). Globally, such restrictions, although effective in limiting COVID-19 transmission, created tensions with industry and the public, lessening the sustainability of such measures (Wood et al., 2022).

The emergence of rapid antigen tests (RATs) for COVID-19 provided a potential tool for systematic surveillance workplace testing. COVID-19 surveillance initially focused on laboratory-based polymerase chain reaction (PCR) testing, which although accurate, requires a day or more for laboratories to process high test volumes (Ingram et al., 2021). RATs are diagnostic tests that detect the presence of viral disease through nasopharyngeal or saliva samples (Augustine et al., 2020). Compared to PCR COVID-19 testing which requires samples to be collected by trained individuals followed by laboratory testing, RATs can be self-administered, performed on site, and generally produce a result in 15 min (Augustine et al., 2020). The trade-off is their lower sensitivity compared to PCR testing (Augustine et al., 2020). RATs have been evaluated primarily within healthcare settings, where medical staff administered the tests to workers with high fidelity to the manufacturer’s protocols (Bond et al., 2022; Muhi et al., 2021; Ventura et al., 2022). However, interest in using RATs quickly emerged in other sectors with high COVID-19 exposure, including education, supermarket supply chains, and the construction industry. However, in Australia and elsewhere, there was little policy guidance or evidence on how to effectively manage the implementation of RATs in the workplace including logistical requirements (e.g., space, infrastructure, regular supply), quality control (e.g., monitoring of adherence to test protocol, data collection, worker education), and strategies to reduce worker resistance.

While studies point to the effectiveness of RATs in detecting positive cases in non-medical settings (Iddins et al., 2021; Orlandi et al., 2021; Papenburg et al., 2022; Rosella et al., 2022; Schulte et al., 2021; Tulloch et al., 2021), the literature offers few lessons regarding how to create enabling conditions for effective and sustainable implementation. Using a case study from Victoria, Australia—where the construction industry was one of the few industries allowed to operate during lockdowns and a key source of COVID-19 transmission (SBS News, 2021)—this study examines the experience of the first large-scale infrastructure project in the state to implement mandatory workplace RAT. Applying the RE-AIM framework, our mixed methods study examined factors associated with the adoption, implementation, and maintenance of this intervention.

## Methods

### Study Setting

Large-scale lockdowns were used to prevent extensive community transmission of COVID-19 in Australia. In the state of Victoria, there were six lockdowns totaling 242 days between 2020 and 21 (Rajagopalan et al., 2022). Except for a 2-week period during the 6th lockdown, construction industries were permitted to work with restrictions on workforce size (Victorian Building Authority, 2021). Australia relied solely on PCR testing until August 2021, when the first RATs were approved for use under healthcare provider supervision. The study site formed part of an industry collaboration with the Victorian Department of Health to pilot workplace use of RAT in the State of Victoria.

The construction site workforce comprised two main teams: (i) a project workforce of around 300 employees that oversaw the project from start to finish and included project managers, health workers, administrators, engineers, warehouse managers, procurement, and communications staff; and (ii) a temporary workforce of around 700 staff on any given day who provided specialized skills (such as construction, engineering) at different points during the project.

Between the 5th of October and the 21st of November 2021, when mandatory workplace RAT was implemented and this study was conducted, COVID-19 prevalence was relatively low; case numbers ranged from ~ 1500–2100 cases per day in Victoria (B.1.617.2 ‘Delta’ variant) among a population of ~ 6.8 million (The Victorian Government, 2022). The surge of primarily Omicron (B.1.1.529) in Victoria peaked at ~ 50,000 daily cases in January 2022 after study completion (Bennett, 2021; The Victorian Government, 2022).

In determining how to roll out the mandatory testing regime, the construction project used several key implementation strategies to support intervention effectiveness and fidelity.

#### Strategies to Support Implementation and Intervention Effectiveness

Strategies to support intervention effectiveness included using a saliva-based test, monitoring and validation of the test result by health workers, and staffing entry gates to prevent workers from accessing the site without a RAT result. The RAT selected for this study was the Ecotest COVID-19 Antigen Saliva Pen Test (Manufacturer: Assure Tech Co. Ltd.: sensitivity: 88.9%, specificity: 98.6%, overall agreement: 96.2%) (Assure Tech Co. Ltd., 2022). This test was selected by the construction company as it was the only saliva-based RAT approved in Australia which, compared to nasal-based RATs, was predicted to be more acceptable to their workers. The manufacturer’s protocol outlines that the applicator be held in the mouth for two minutes, then returned to the extraction buffer cap. The interpretation of the test strip result should be performed between 15 and 30 min after completing the test (Assure Tech Co., Ltd. 2022).

From 5th of October 2021, worksite management required each worker to present a completed RAT which showed a negative result before entering the site at the beginning of each shift. After which, the RAT was disposed of in a waste disposal bin. If a worker chose not to perform a RAT, they were prohibited from entering the site and unable to work until a test was presented. In line with government policy, which outlined that results were required to be validated by a healthcare provider, the construction company sub-contracted health workers from an external organization who were responsible for viewing and interpreting the test result. If a positive (‘non-negative result’ as termed by the worksite) was presented, the individual was directed to call their supervisor or a safety officer from their car and then do a PCR test at a government testing facility. For invalid tests, the individual was required to redo the RAT.

#### Implementation Strategies

Strategies put forward by the construction project to support implementation included providing information about the testing regime to workers on a recurrent basis, supplying RATs to workers in advance of their next shift, and bolstering the health workforce with COVID-19 marshals.

Training on how to perform a RAT was offered on a recurrent basis given the high turnover of staff across different shifts. At the beginning of the implementation period, workers were shown an instructional video at all-staff meetings at shift commencement (‘toolbox pre-start meeting’). For workers who commenced work after the implementation period, information was mandated as part of their onboarding training. As there was no consistent training strategy throughout the implementation period, there was no systematic way to measure adherence to training. The construction site operated on a 24-h a day basis in two 12-h blocks. There were approximately 300 full-time staff and many contractors, amounting to approximately 700 workers on each 12-h shift, with significant day-to-day turnover in contractors.

To help reduce testing bottlenecks at the beginning of each shift, workers were supplied with a RAT to take home the night before. However, those attending their first shift received the test when they arrived on site. Further, the company trained a number of construction workers as COVID-19 marshals to assist the health workers in handing out tests and managing workers passing through the entry gates.

### Study Approach

In this context, we investigated:

- What factors influenced the adoption of RAT in a large-scale construction project?
- What factors affected fidelity to the testing regimen by the workforce?
- What factors impacted intervention maintenance over the course of the project and over the longer term?

#### Sampling, Recruitment, and Data Collection

We used a convergent mixed methods study design to concurrently collect qualitative and quantitative data through surveys, interviews, and onsite observation (Fetters et al., 2013). A concurrent approach was used given the short construction phase (6 weeks, 5 days), to gain implementation insights using multiple sources, and to compare policies, attitudes, and perspectives with on-ground practice.

All management staff were invited to semi-structured interviews which were conducted between the 1st and the 11th of November 2021 (week 4 and 5 of implementation). Interviews inquired about the company’s motivation to use RATs, the planning process, communication with workers, logistical issues, and perceptions of worker attitudes, compliance, and sustainability. Interviews with onsite workers and health staff were conducted opportunistically during break times. Interviews inquired about communication of RAT procedures, worker attitudes, challenges, and views on home-based testing. Interviewees received a $20 grocery store voucher for their time. Written consent to participate and record the interview was obtained by all participants. Interviews were conducted by five members of the research team on two separate occasions on the 31st of October and the 7th of November (week 3 and 4 of implementation), with debriefing at day’s end to identify emergent themes and findings. Interviews were audio-recorded and transcribed using an automated transcription service (otter.ai). NC de-identified all transcripts and reviewed their accuracy.

Second, a survey was sent to workers which comprised primarily closed-ended questions about attitudes to testing, protocol knowledge, practices relating to the last time that they took a RAT, and challenges encountered. During week one, the worksite sent workers an SMS with the online survey link (Qualtrics). Survey respondents could enter a prize draw to win one of three $100 gift vouchers. Due to the high-volume worker turnover, a repeat invitation was sent in week two.

Third, an observation checklist was developed to observe intervention fidelity to testing protocols. Observations involved one researcher positioning themselves at one of three site entry points and observing the process of people returning a negative RAT before gaining entry at the beginning of the shift. The researcher noted their observations over a period of between 10 and 30 min. This included observations on the estimated number of people returning negative and non-negative tests, the presence of health workers overseeing and COVID-19 marshals supporting testing, the presence of measures for infection control (e.g., social distancing), and the appropriate disposals of completed tests. When possible, the researchers also observed the compliance of people performing a RAT to the testing protocol. Six observation checklists were completed by five researchers on two separate occasions (31th of October & 7th of November 2021—week 3 and 4 of implementation). Observational data were primarily collected for validation purposes to observe if there were significant discrepancies between the experiences reported in interviews and surveys and actual practice on the ground. Company registers documented the number of RATs and referrals for PCR over the 7-week construction project (5th of October–21st of November, 2021). Table 1 provides an overview of the data collection method, target sample, recruitment strategy, and topics covered for each data source. The interview guides, survey questionnaire, and observation checklist are included as supplementary files.

**Table 1:** Overview of data sources

Data source	Target population	Recruitment	Topics covered	RE-AIM component targeted
Interview (semi-structured)	Administrators, Managers	Referral	• Company’s motivation to use RATs • Preparatory activities • Communication with workers • Implementation perspectives (successes, challenges) • Perceptions of worker attitudes and level of compliance • Perspectives on sustainability	Adoption Implementation Maintenance
Interview (semi-structured)	Workers (construction, health workers)	Convenience (present in break rooms)	• Attitudes to testing • Protocol knowledge and practices • Communication with workers • Challenges • Perspectives on sustainability • Views on home-based testing	Adoption Implementation Maintenance
Survey (primarily closed-ended)	Workers (construction, health workers)	By SMS from construction project	• Attitudes to testing • Protocol knowledge and practices • Communication with workers • Challenges	Adoption Implementation
Onsite observation (checklist)	Workers (construction, health workers)	Convenience (present on day)	• Overall compliance of the site entry to implementation protocol (e.g., presence of health workers overseeing and COVID-19 marshals supporting testing, social distancing, appropriate disposals of completed tests) • Worker compliance to RAT testing protocol	Implementation

#### Analysis

Our analysis was guided by the RE-AIM framework (Glasgow et al., 1999). We focused on the latter three domains (adoption, implementation, and maintenance) which aligned with our focus on understanding feasibility, acceptability, and sustainability of the RAT regime. As all participants were employees, we discuss willingness to participate under the adoption domain rather than reach as done elsewhere (Bethany et al., 2019).

We used a manifest analysis approach to qualitative content analysis (Hsieh & Shannon, 2005). In Excel, we deductively coded text to the three RE-AIM domains of interest: adoption, implementation, and maintenance. We then inductively coded factors relevant to these domains, e.g., attitudes related to acceptability (positive, negative); factors affecting intervention fidelity (adherence to protocol); and short- and long-term perspectives on sustainability (positive, negative). One author led the primary coding. The coding framework was informed by key insights which emerged from the end-of-day debriefing meetings conducted after each day of interviewing. The senior authors reviewed the coding framework, and a second author reviewed all transcripts against the primary coding. Given the straightforward content of the interviews, no major discrepancies were raised. To support trustworthiness and dependability of the thematic analysis, the key themes were discussed with the research team, most of whom had conducted interviews, to achieve consensus. During these discussions more minor themes were excluded. For the quantitative analysis, de-identified survey data were exported from Qualtrics to Stata 16. Lastly, observation data were reviewed by the author who reviewed transcripts against primary coding in order to determine whether what was observed was corroborated within interviews.

Qualitative, quantitative, and observational data were integrated using an *integrating through narrative approach*, in which different types of data are presented together on a theme-by-theme basis, allowing for mutual validation and convergence of results (Fetters et al., 2013). The qualitative data, which comprehensively covered the full range of intervention participants (administrators, managers, construction workers, health workers), were used to generate the primary themes. Our survey data were more limited, in terms of a low response rate and the poor coverage of construction worker perspectives. Therefore, the quantitative data were used as supporting evidence and presented in relation to the qualitative data. Observational data are presented briefly for validation purposes.

## Results

Over the seven-week mandatory RAT period, 37,720 RATs were conducted on site [67 non-negatives identified (0.18%)]. Only one ‘non-negative’ RAT result was confirmed PCR positive. In total, 40 construction workers, six health staff, and five managers and administrators were interviewed, 30 workers completed surveys and nine workers were directly observed completing a RAT. Brief participant characteristics are provided in Table 2, noting that characteristics of workers are not available for direct observations. Of those surveyed, over three-quarters (77%) reported that they had conducted between 6 and 10 RATs at the time of survey completion.

**Table 2:** Characteristics of interviewees and survey respondents

Participant characteristics	Interview	Survey
	*n* (%)	*n* (%)
Total	51	30
Gender^#^		
Male	41 (80.4)	21 (70.0)
Female	7 (13.7)	9 (30.0)
Age group in years^#^		
18–29	12 (23.5)	8 (26.7)
30–39	7 (13.7)	10 (33.3)
40 +	5 (9.8)	12 (40.0)
Role at construction site		
Construction	38 (63)	7 (23.3)
Administration	3 (6)	11 (36.7)
Health staff	6 (12)	–
Management	5 (10)	7 (23.3)
Other +	–	5 (16.7)

Different symbol indicates communications, engineering, procurement, warehouse management

^#^Not all demographic characteristics were reported by participants

### Adoption

**Fig. 1:**
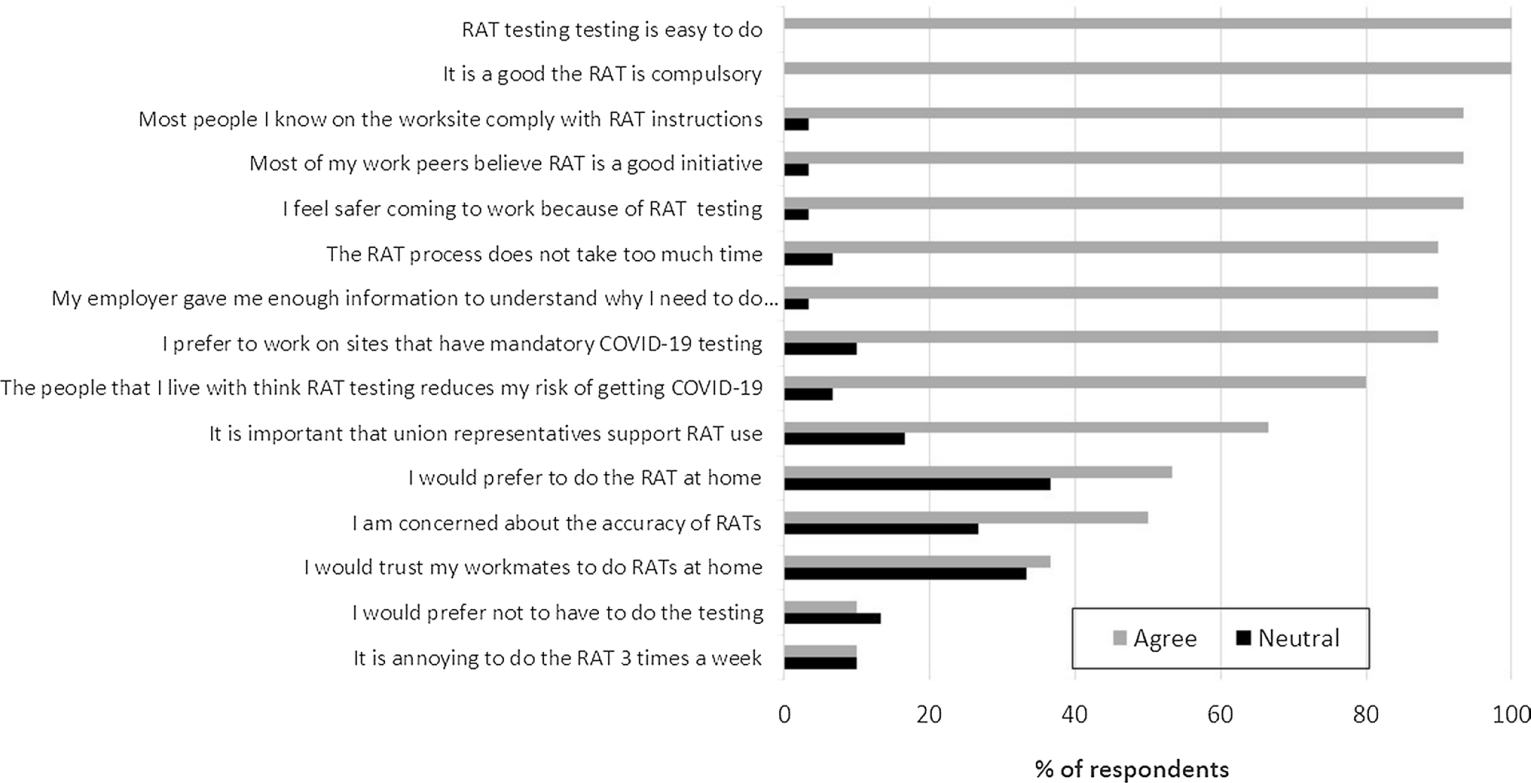
Survey respondents’ attitude towards the RAT protocol

Viewed as a time-limited mandate, there was a high degree of acceptance for RAT as a condition of worksite entry by managers and the workforce, with little pushback observed. Key reasons for high acceptability included the desire to keep jobs going, ease of test-taking, and, for some, increased safety and the peace of mind afforded by RATs. Workforce quotations underscoring themes elaborated below are presented in Table 3 and survey responses in Fig. 1.

**Table 3:** Illustrative Quotes Related to Adoption

Sub-theme	No	Illustrative quotation
Minimizing site closures and keeping jobs going	1.1.1	Interviewer: And what's your understanding of why people at your workplace are being tested regularly at work? Respondent: So we can remain open. Yeah (Worker)
	1.1.2	So we had a couple of cases before the lockdown…there were a couple of guys on site that had COVID at different times and because of that, a lot of people lost work, like we had to go back home, get tested and isolate (Worker)
Simple testing procedure	1.2.1	Yeah, pretty easy. Yeah. Straight in the mouth…Sort of sucking on a lollipop and then straight on to the test (Worker)
	1.2.2	When we saw or when we discovered the saliva testing kit, it lent itself to be more efficient method of mass testing before work and essentially able to be administered by the workers (Manager)
Safety and test accuracy	1.3.1	Interviewer: And do you think that it [RATs] does make the site safer? Respondent: I don't know. I'm assuming it does. Yeah. But I don't know. Because they were saying that they're [the RAT tests] only 50% accurate. So that is a 50% chance…That leaves a bit of room for error (Worker)
	1.3.2	you've got to make sure the quality issue is there. If the test has to be in your mouth for two minutes, you hear different people and interpretations about whether it's 90 s, 60 s too much fluid, not enough. If you've had protein, if you've had a coffee, if you've done this… (Worker)
	1.3.3	[RAT] is a good investment, but yes, for us as an alliance and as a business, it’s just thinking outside the box, being ahead of the game, looking at ways to maintain peoples’ health and safety when they come to work. So it’s all part of the health and safety…to provide a safe workplace (Worker)

#### Minimizing Site Closures and Keeping Jobs Going

The workforce clearly understood that RAT was a requirement of going back to work (Quote_1.1.1), voicing that RAT was a way to ‘*just to be able to get back to work’*’ and workers felt ‘*pretty well excited’*’ to be back. Workers gave examples of having to miss shifts in the past due to positive cases in their team and government policy requiring all contacts to isolate (Quote_1.1.2). Given that it was a critical infrastructure project with strict deadlines, it was considered a ‘*good investment*’ by management. From the managers’ perspective, high acceptability of RATs was influenced by the fact that after implementation, the construction project did not experience any COVID-19-related disruptions by way of site shutdown or large numbers of staff being furloughed as close contacts. Other factors which may have supported workforce acceptance of RATs included the backing by their union, as noted by 67% (*n* = 25) of survey respondents.

#### Simple Testing Procedure

The ease of taking the RAT contributed to the test’s acceptability. Among interviewees, the saliva-based RAT was seen as simple, non-invasive, and reasonably easy to incorporate into daily workflows (Quote_1.2.1). Managers viewed the selection of a saliva-based versus a nasal-based test as important for implementation feasibility (ability of workers to self-administer) and worker acceptability (Quote_1.2.2). All survey respondents similarly reported that doing the RAT was easy and expressed minimal challenges. Remembering to pick up the test (13%, *n* = 4), waiting the recommended amount of time for test results (6.7%, *n* = 2), and extra shift time to do the RAT (6.7%, *n* = 2) were the most commonly reported challenges among survey respondents.

#### Safety and Test Accuracy

From a management perspective, implementing RATs was considered an appropriate workplace measure that helped to contribute to a safe working environment and a return of their workforce (Quote_1.3.3). However, interviewees and survey respondents held mixed perceptions about the degree to which RATs improved workplace safety. Many interviewees indicated that the RATs provided ‘*peace of mind’*’ for themselves and their family. At the same time, there was considerable uncertainty around the extent to which the tests truly made them safer; among some, the RATs were believed to be only 50% accurate, leaving considerable ‘*room for error*’ (Quote_1.3.1). Workers believed that test results could be affected by many variables, including amount of saliva and what they had to eat or drink prior to the test, which in turn contributed to a high number of false positives (Quote_1.3.2). These mixed sentiments were echoed among survey respondents: more than 90% indicated that they would prefer to work in settings where RATs are used, felt this preference was supported by their co-workers, and believed most workers complied with RAT protocols. Nearly all survey respondents believed the tests made them and their families feel safer (80%), yet half (50%) held doubts about the test’s accuracy.

### Implementation

#### Communication and Worker Knowledge

Interviewees and survey respondents indicated that the source and quality of information received varied among workers. According to interviewees, some received information in pre-shift meetings, others received links to informational videos and some only received information upon first entry. Among survey respondents, instructional information was mostly received verbally (91.3%, *n* = 21) alongside video (52.6%, *n* = 10) and written (47.7%, *n* = 10) instruction. Instructional information was received from onsite health staff (35%, *n* = 8), onsite COVID-19 marshals or safety team (26%, *n* = 6) co-workers (17%, *n* = 4), and from their manager or administrator (17%, *n* = 4). Most survey respondents (92%, *n* = 23) correctly identified a negative RAT result.

#### Intervention Fidelity

The interviews, observations, and surveys all revealed gaps in adherence to testing protocols. Misconceptions and logistical constraints were key factors that influenced adherence. Workforce quotations underscoring themes related to intervention fidelity are presented in Table 4.

**Table 4:** Illustrative quotes related to Implementation

Sub-theme	Ref	Illustrative Quotation
Adherence to protocols	2.2.1	It's been a journey. Certainly in the early days of the commencement of the testing regime, worker education was an area for improvement so that every time we had new workers or even just existing workers, first few days, was a learning process … Using the test kits incorrectly e.g., not removing the protective cap from the swab, or even returning the protective cap from the swab and then proceeding to try and join the swab with chemical reservoir. So we have seen, certainly an improved user skill. But always, you know, first time users, its worth taking the time and that's the benefit of having the life aid paramedics on site at those control points to provide instruction for first time users (Manager)
Monitoring entry	2.3.1	The early phase planning was going to try to see how we can manage the large number of four groups, we're talking about 600 people almost on average per shift, and we run day and night shift. The main idea was to make sure that we control the mass (Manager)
	2.3.2	Probably a more effective method would be to have the workers conducting the tests in a controlled environment at our pre-start, that would give us high efficiency and competence that tests are being conducted correctly and for the right duration, etc. But that would expose us to being a close contact site if we had a positive worker in the pre-start compound or in the pre site area. So we looked at… we had to assess the benefits and risks of allowing workers to test outside of the worksite. So essentially, take a test home every day, and tests on the way to work, but no later than before the entry to the worksite (Manager)

Despite general perceptions among workers that the test protocol was straightforward and relatively easy to comply with, managers and health and safety staff held mixed views regarding workers’ compliance. Some managers reported several challenges in the ‘*early days*’ related to ‘*user skill*’ and failure to follow the instructions, while other managers felt that worker compliance was generally high (Quote_2.2.1, Table 4). In the context of significant workforce turnover, several managers and health and safety staff raised compliance issues related to the quality and consistency of information for onboarding new workers.

##### Actions Following Non-negative Results

A health worker noted that diversity of professionals (nurses, paramedics) and non-professionals (COVID-19 marshals) employed to assist with the testing, and differences in their level of training, knowledge, and time on site, led to different interpretation of the protocols following a non-negative result. Numerous interviewees believed that they should re-test following a non-negative result because of faulty kits and false positives due to consumption of certain foods and drinks (e.g., coffee). Some confusion was similarly echoed in survey respondents. Five survey respondents reported non-negative or invalid results. Actions following these results included performing another RAT, completing a PCR test, and contacting their manager. Most survey respondents were aware of the correct actions following a non-negative result, indicating they would stay in their car and call someone (70%, *n* = 16) and/or do a PCR test (57%, *n* = 13). However, one survey respondent with an invalid result indicated that they were “not entirely sure if my single line tests were invalid” and “never made aware there was a difference in single line results” *(Worker).*

##### Time to Results

Interviews revealed several misconceptions. Regarding time-in-mouth, it was commonly believed that 30 seconds was sufficient to get enough saliva and that waiting longer would produce false results. For time-to-results, workers generally perceived that the test was readable after the control line appeared in the first couple of minutes (even among those who recalled the instructions to wait 15 min). Further, some perceived the 15-min wait as ‘*annoying*.’ Over a quarter of survey respondents (*n* = 7) indicated they did not hold the test in the mouth for the full 2 min and few waited the full 15 min for test results according to the manufacturer’s instructions (Table 5). Our observations further noted that eight of nine (89%) of those observed did not follow the prescribed waiting time to result. Variables, such as the diversity of staff involved in implementation and differences in the level of training and information provided to workers, influenced implementation fidelity (i.e., the degree to which strategies that support the implementation of an intervention were used as intended). Inconsistencies in implementation fidelity likely impacted intervention fidelity (i.e., the degree to which the innovation was implemented as intended) as shown by the variations in actions taken following non-negative results and differences in the way tests were taken relative to the manufacturer’s instructions.

**Table 5:** Adherence to testing protocols at the most recent rapid antigen test among survey respondents

Survey question	Response	*n* (%)#
Total		30
Last rapid antigen test was conducted	In the car	17 (68.0)
	Worksite entry	1 (4.0)
	At home	7 (28.0)
Last rapid antigen test was conducted alone	Yes	20 (80.0)
	No	5 (20.0)
Last rapid antigen test; time to hold pen in mouth	< 30 s	1 (4.0)
	One minute	4 (16.0)
	Two minutes	18 (72.0)
	3 to 5 min	2 (8.0)
Last rapid antigen test; time to wait for test to finish	Until the control line appeared	6 (24.0)
	< 5 min	5 (20.0)
	5 to 10 min	7 (28.0)
	15 min	4 (16.0)
	> 15 min	3 (12.0)
Last rapid antigen test; used timer when doing the test	Yes	9 (36.0)
	No	16 (64.0)

^#^Denominator is not always the same because of missing data (25 respondents completed the questions about protocol adherence at their last rapid antigen test)

##### Monitoring Entry

Given the hundreds of workers entering the site each shift, a major preoccupation by managers and health workers was the challenge of limiting large numbers of workers from congregating at the front entrance while awaiting test results which would create a potential COVID-19 transmission risk (Quote_2.3.1, Table 4). Our observation found that workers seeking to collect tests contributed to crowding at the entry gate alongside workers waiting for test results. Reasons for testing at the front gate included new workers who had not received any test kits, workers who had not received a kit to take home after their previous shift due to stockouts or had forgotten them, and preference to not wait in the car. Health workers indicated they had varying success in requesting workers to move away (e.g., take a walk). In response to crowding at the entrance, site management adapted the protocol, advising workers to conduct the test while driving to work so that the result was ready upon arrival. This was done to improve feasibility while acknowledging the limitations on quality assurance (Quote_2.3.2, Table 4). Managers expressed a high degree of trust in their workforce to appropriately take the test on route without direct supervision. Workers interviewed indicated that testing while traveling on route to the site was convenient and did not waste their time. Overall, 68% (*n* = 17) of survey respondents reported conducting their most recent RAT in their car.

### Maintenance

While the cost and resources to implement RATs in the project were significant, the project was willing and able to absorb these costs. In contrast, waning worker acceptance over time emerged as a challenge to sustainability. Workforce quotations underscoring these themes are elaborated in Table 6.

**Table 6:** Illustrative quotes related to maintenance

Sub-theme	Ref	Illustrative quotation
Cost	3.1.1	On some rough numbers I heard yesterday, we've avoided costs in the order of $6 million by doing RAT, that we would have incurred if we had to shut down aspects of the site, because of people being otherwise close contacts – Manager
Worker acceptance	3.2.1	I think, personally, now that we're up to 80% [vaccination rate of the adult population]. I don't think it's feasible [mandatory workplace testing] … We need to start moving on… I think if the state's 80% vaxed, you've only got a 20% window, I don't see a big issue – Worker
	3.2.2	There's no remuneration due to that either. It's sort of I just find it a bit unfair, really, especially… something that's not as accurate as it should be when it is concerning people's livelihoods—Worker
Home-based testing	3.3.1	I'd be worried about people fudging it, getting someone else to do it and then bring it in. But I really like the convenience factor of me being ready to give it to them when I get to the gate

#### Cost

The costly investment in RATs was perceived by managers as worthwhile, as internal projections estimated that using RATs saved the company millions by avoiding costly shutdowns and project delays due to COVID-19 (Quote_3.1.1).

#### Worker Acceptance Over Time

The overwhelming sentiment among workers was that double vaccination already conferred significant protection and additional testing was not needed, especially given uncertainty around the accuracy of RATs. As such, the majority indicated that they ‘*felt safe’*’ and did not think regular testing would be useful or worth the cost once the State of Victoria achieved its vaccination targets (Quote_3.2.1). Some workers were uncertain about the payment policies for missed shifts, including for casual workers, and heard rumors about workers missing out on double shifts because of false positives and felt it was unfair to lose days of work based on inaccurate test results (Quote_3.2.2). Others raised the ‘*annoyance*’ of continuing to do daily tests and ‘*running out of patience*.’

#### Home-based Testing

During the implementation period, the Australian government approved several home-based COVID-19 testing kits (Therapeutic Goods Administration, 2022b). Workers interviewed relayed advantages of potentially shifting to home-testing, including convenience and ease, but many had reservations about workers falsifying tests (Quote_3.3.1). While 53% (*n* = 16) of survey respondents would prefer to do home-based testing, only a minority (30%, *n* = 9) felt they could trust their workmates to appropriately take the tests at home.

## Discussion

In the context of rapid approval of and implementation of RATs as a COVID-19 risk mitigation measure in Australia and globally, this study provides important implementation lessons on factors affecting the adoption, implementation, and maintenance of mandatory RAT in a large workplace within a low prevalence setting. In the Australian context, following months of significant disruptions to industry operations due to COVID-19-related lockdowns, our study found high acceptance among workers and managers for mandatory RAT in the short term. Factors which supported adoption by workers included the desire to stay in work, the perception that the testing procedure was easy and non-invasive, and a sense of workplace safety. These factors are consistent with other evaluations of RAT usage in the workplace (Doron et al., 2021; Nguyen et al., 2022). For managers, economic considerations were forefront in supporting adoption. Mandatory use of RATs made sense economically, preventing closures and disruptions. However, in implementing the testing regime, implementation and sustainability challenges emerged related to knowledge and adherence to testing protocols; managing crowding among workers; and maintaining worker acceptance in the context of high population vaccination rates.

The policy environment around COVID-19 testing in Australia shortly before and around the time of this study was rapidly changing. In August 2021, the Therapeutic Goods Administration first approved COVID-19 RATs for use only under the supervision of a trained healthcare provider (Therapeutic Goods Administration, 2022a). Prior to this, Australia had solely relied on PCR-based COVID-19 testing, meaning there was minimal community literacy or experience with RATs at the time of implementation. This may have contributed to the varied adherence to RAT protocols observed within this study. Consequently, all education on rapid antigen testing was reliant on worksite-provided education. Other studies have identified similar challenges related to poor adherence. A UK study found that only 8.6% and 25.3% of staff achieved a protocol adherence rate of > 75% and > 50%, respectively, leading to no reduction of COVID-19 outbreaks (Tulloch et al., 2021). Poor adherence to testing procedures may lead to increased frequency of false negatives with the potential for workplace outbreaks, ultimately undermining costly investments in mandatory surveillance RAT regimes (Paltiel et al., 2021). To promote stronger intervention fidelity, our study suggests that strategies to improve implementation fidelity—such as provision of consistent messaging to promote compliance to testing protocols—is important. Attention to consistent and effective implementation strategies is especially important in workplaces with high staff turnover.

While insufficient intervention fidelity (adherence to protocols) can diminish effectiveness, the implementation literature also shows that insufficient room for adaptation can hinder uptake, sustainability, and effective implementation (Hawe et al., 2004; Pinnock et al., 2017). Unlike many health interventions which are designed and optimized in a research setting, the construction project had a significant role in determining the intervention design and implementation strategies, and by extension, significant autonomy around adaptation. Limited space and the requirement to have trained health personnel monitor worker testing also emerged as a significant logistical challenge. In our study site, managers responded by adapting the testing protocol and encouraging workers to take the test in their cars enroute to work. This adaption may be insightful for other workplaces considering validating RATs upon an employee’s arrival on site, which are likely to contend with similar constraints. While this adaptation diverged from government policy around health worker-observed RAT, it improved feasibility; thus, highlighting tensions between best practice (government and manufacturer guidelines) and implementability. Similar challenges were observed in a large-scale implementation of RAT in the workplace in Canada, where the implementation approach was also adapted to allow home-based testing (permissible according to Canadian guidelines (Rosella et al., 2022). Site adaptability to the implementation of surveillance testing is critical to implementation success, and has been shown in the implementation literature to be essential for making new ways of working part of regular practice and supporting sustainability in the longer run (Chambers et al., 2013). The agility of the United Kingdom logistics sector in implementing measures to protect their staff and business, often in a context of limited Government and policy guidance, was an important component of their response to COVID-19 (Wei et al., 2022). There needs to be flexibility in systems to allow for modifications and adaptions to enable implementation success of surveillance testing, particularly, in rapidly evolving health and policy landscapes. Importantly, adaptations also need to be quality assured and monitored to ensure that changes made on the fly improve the fit and/or effectiveness while also complying with the current policy environment. In assessing interventions, the use of an implementation framework to guide the approach where adaptations are applied can help to both strengthen the intervention fidelity (the degree to which the intervention is implemented as intended) and implementation fidelity (the degree to which strategies that support the implementation of an intervention were used as intended). This, in turn, is likely to lead to better outcomes and the sustainability of implementation (Albrecht et al, 2022).

While rapid testing was supported by the workforce to keep the worksite open and safe, our findings foreshadowed significant challenges in maintaining ongoing support in the long term with implications for the sustainability of mandatory testing as a condition of work. This study was conducted during a unique timepoint when the Australian state of Victoria was nearing pre-determined COVID-19 vaccination targets and still had low community prevalence. Under the national plan, lockdown restrictions would be eased progressively when 70% and then 80% of people over 16 years of age were double vaccinated (The Australian Government, 2021). The vaccination policy, coupled with rising population vaccination rates, created a sense of optimism that vaccination would allow for the safe opening of society. This likely influenced workers’ acceptability of mandatory testing as a time-limited measure. As seen in Australia and internationally, there has been a generalized decrease in support for ongoing mask, vaccination, and testing mandates, with many governments subsequently lessening and/or abandoning these requisites (International Monetary Fund, 2021). While many countries, including Australia, have now approved home-based testing, voluntary testing requires public willingness, access to tests, and tracking systems to support surveillance efforts—features with which many countries and organizations are struggling (Nguyen et al., 2022). Greater guidance from policy makers about how to adapt testing protocols to be appropriate for the background COVID-19 prevalence may help maintain levels of acceptability among staff, as has been noted elsewhere (Nguyen et al., 2022).

## Limitations

Given the limited focus on the implementation experience of RATs, compared to studies reporting on RAT effectiveness in case detection, this study provides important findings regarding adoption and implementation. Our use of direct observation combined with interviews provided value in confirming reported practice with actual practice. However, the low number of responses to our survey is a major limitation to our quantitative data, particularly that only seven surveys were completed by construction workers. Several factors may have contributed to this. The interviews were conducted at a similar time to when the survey was open, with all interviewees receiving a $20 voucher compared with an opportunity to win one of three $100 vouchers for survey completion. Possibly, workers preferred the certainty of a $20 voucher. Furthermore, the interviews were conducted face-to-face. It is possible that workers were more likely to participate in an interview when approached directly rather than remembering to complete a survey. While our study was conducted in a single, for-profit worksite, observed technical challenges (e.g., protocol adherence, managing logistics) are likely salient to other workplaces. However, the impetus for adoption may be highest in for-profit industry contexts, where delays and shutdowns due to workers contracting COVID-19 creates immense cost in lost work and challenges in managing on-time project completion. Supplying daily tests to workers may be prohibitively expensive in non-for-profit and public sectors, and in low-income country settings. Further, the high level of worker acceptability that was observed may have been influenced by the particularly restrictive lockdowns in Australia. This study was performed in a setting with a relatively low prevalence of COVID-19 and high vaccination coverage. Future studies should investigate how implementation and sustainability may differ in contexts of high compared to low COVID-19 prevalence and how testing regimes could be adapted for implementation in other industries and public sectors where budgets and tight deadlines may be less salient.

## Conclusion

Achieving intervention fidelity and implementation fidelity in a rapidly changing context is difficult, particularly so during a pandemic. Using implementation tools, such as the Plan, Do, Study, Act Cycles (PDSA), can create space for adaptations to be considered as part and parcel of implementation and provide for systematic assessment of these adaptations (Institute for Healthcare Improvement, n.d.; Gilbert et al, 2020). While these tools are more commonly used in health and academic settings, they may be less familiar to commercial and non-profit enterprises—supporting their use in these settings may be beneficial to the sustainability of new interventions implemented in fast-moving environments.

With COVID-19 likely to continue to circulate for years to come, finding ways to keep workplaces open while keeping workers safe will be a priority for countries and businesses. The implementation of RATs on a large-scale construction site in the State of Victoria, Australia was an acceptable and feasible short-term measure to keep worksites open by minimizing COVID-19 transmission, while acknowledging issues with adherence to the testing protocol. As use of RATs becomes normalized and increasingly non-mandatory, new strategies will be needed to maintain worker willingness and quality control over results. Further, developing centralized systems for recording both home and workplace testing results will be important for surveillance and vigilance among the population.

## Supplementary Information

Below is the link to the electronic supplementary material.

Supplementary file1 (DOCX 44 KB)
