# Supplementary material for: The Implementation Experience of COVID-19 Rapid Antigen Testing in a Large-Scale Construction Project in Victoria, Australia
Source: Glob Implement Res Appl. 2023 May 30:1–13. Online ahead of print. doi: 10.1007/s43477-023-00085-4 (PMC10228896; doi:10.1007/s43477-023-00085-4)
Supplement: Supplementary file 1 — Supplementary file1 (DOCX 44 KB) [file 43477_2023_85_MOESM1_ESM.docx]

**Manager interview guide**

**Introductory questions**

What’s your name?

What’s your role?

How long have you been working on the <name> project?

**Background to RAT**

Can you tell me a bit about how it’s been managing construction projects through COVID?

Have you had to deal with a shutdown?  If so, what was that like?  What were the impacts/consequences?  What are the costs for the company?

Can you tell me about how you came to the decision to implement RAT?  Whose idea was it initially?  How was the decision made?  What factored into that decision?  What other options were considered?  When was the decision made?

Why was the decision made to make it mandatory?

What kind of support did you receive to make this decision, and from whom?

How was the union involved?  What was their reaction?

Were there any other organisations involved?

Have you spoken to any other projects/industries about their experiences implementing RAT?  Were there experiences relevant?  What did you learn from them?

What are the main outcomes you expect to see from RAT?

**Change management**

Once the decision was made to implement RAT, how was this communicated to the workers?

- Who communicated the decision and when?
- What communication materials were used?
- What was the messaging?

What kind of planning had to be undertaken for RAT to begin?

What have been the major changes from the first trial to how the testing is conducted now?

**Workers’ attitudes**

From your point of view, what’s been the workers’ reactions to having to take the test?  Has there been any pushback?

If so, what was their complaint?

If not, why do you think they’re happy to take the test?

Do you think there are any workers that have skipped coming in to work because of having to take the test?

**Compliance**

From your perspective, do you think the workers have a good understanding of the testing protocol?  If not, why not?  If so, what do you think has contributed to that?

What happens when a ‘non-negative’ or invalid result comes up?

Have there been any positive cases detected so far (confirmed through PCR)?  If so, what happened?

Have there been any issues in workers adhering to the testing protocol?  If so, how have they been managed?  How frequently do they occur?

Prompts:

- Waiting in the car and calling the safety officer if they have a ‘non-negative’ or invalid result
- Waiting 15m to read the results

What about instances in which the health workers don’t adhere to the protocol?

Prompts:

- Allowing multiple tests if they have a ‘non-negative’ result
- Getting out of the car to inform staff if they have a ‘non-negative’ result

**Logistics**

How have the logistics of the testing been working?  What have been the challenges?  Is there anything that you think could be improved?

Prompt:

- Has there been any issue with having only the one entry/exit?
- Does it get crowded in the entry area?
- Is there a possibility of someone being able to get through without showing a test?
- How have positive/negative antigen tests been recorded?  Positive/negative PCR tests?

Have there been any challenges for the health staff in conducting the tests?

Have there been any issues with safety for the health staff?

**Sustainability**

Do you think there will be any issues in asking workers to continue taking rapid antigen tests into the future?

Is there anything you think needs to/should be improved for conducting RAT at projects in future?

Do you think there are any potential ethical or legal issues that could come up because of the testing?

*Towards end of the project:*

Approximately how many (confirmed PCR) positive cases were there?

How many false positives were there?

Are these rates acceptable to the project?

Have the rates of false positives resulted in reduced trust/compliance?

Have false positives had negative impacts on the workers (e.g. loss of income)?

**Alternative arrangements**

There’s been some discussion about having workers take the test at home.  Do you think this would be a good idea?  Why or why not?

Are there any issues or potential problems that we should think about before having at-home testing?

How would the results and quality be monitored?

**Ending the interview**

Is there anything else you want to tell me about the rapid antigen testing that we haven’t already talked about?

**Construction worker interview guide**

**Introductory questions**

How long have you been working in the construction industry?  What’s your role?

Can you tell me your age?  (It can be approximate, if you prefer)

Do you speak a language other than English at home?

Are you Aboriginal and/or Torres Strait Islander?

Are you a contracted worker or work full-time for <organisation>? / What is your employment arrangement i.e. casual, part-time, full-time, contracted?

Do you only work at this site <insert site name> or multiple? If multiple, which other sites have you worked at?

**Change management**

When were you first told about the rapid antigen testing? How was it explained to you?

Did you have the opportunity to ask questions if you wanted to?  [Did you ask any questions?  What questions did you ask?]

[If EAL, Did you get information in your first language?  If not, do you think it would have been helpful?  In what way?]

What is your understanding of why people at your workplace are being tested regularly at work?

Did you have any issues with having to take the test?

If yes, what issues?  Did you raise the issues with anyone? If no, why not?

Do you think the test should be mandatory?  Why or why not?

**Taking the test**

Can you explain to me the process for taking the test?

How does taking the test fit into your daily work routine?  Is it easy or convenient to do the test?  Challenging or annoying?  Can you tell me about what aspects of the process make it easy or convenient/challenging or annoying to regularly do rapid antigen testing at the construction site?

Have you ever had a ‘non-negative’ or invalid result? If yes, what happened?  If no, what would you do if you did?

How would you/did you feel if/when you got a positive result?  What about if you got a positive result that then ended up being negative?

Could you describe your feelings when you first started doing the test?  Has this changed over time?

Would you recommend any changes to the process?  If so, what changes would you recommend?

**Sustainability**

Do you think there will be any issues in asking workers to continue doing rapid antigen testing into the future?

Do you feel that workers having to do the test makes the site safer?

Would you prefer to work at a site that did or didn’t have mandatory testing?  Why?

Would you feel less safe at a site that didn’t have mandatory testing?

**Alternative arrangements**

Have you had a COVID swab test before [where they swab in your nose and throat]?  If you have to take a COVID test to come to work, which do you prefer, the rapid test or the swab test?

There’s been some discussion about having workers do the test at home.  Do you think this would be a good idea?  Why or why not?

Are there any issues or potential problems that we should think about before having at-home testing?

**Ending the interview**

Is there anything else you want to tell me about the rapid antigen testing that we haven’t already talked about?

**Extra questions for COVID Marshals** [They support workers to follow [COVIDSafe principles](https://www.coronavirus.vic.gov.au/six-principles-covidsafe-workplaces) and look for ways to continually improve a business’s COVIDSafe Plan, including entry screening, physical distancing, personal protective equipment (PPE), workplace bubbles, hygiene, cleaning and record-keeping]

Were you provided any training for this role? (It’s called the ‘infection control awareness training’) 
Do you know who to report to or communicate with if any issues arise?

Is your scope of work manageable? (In terms of the number of workers they need to supervise within a geographic area)

How do you receive communications about changes to practice or guidelines? Is it in writing or verbal? How do you communicate changes to other workers, if any?

**Survey questionnaire**

**Evaluation of Rapid Antigen testing at Site**

**Demographic information**

What is your age (in years)?

What gender do you identify as?

- Male
- Female
- Non-binary
- Prefer not to say

What is the postcode of your home address?

What is your country of birth?

- Australia
- Other (please specify)

Which language do you mainly speak at home?

- English
- Arabic
- Mandarin
- Italian
- Greek
- Vietnamese
- Hindi
- Other (please specify)

Do you identify as being of Aboriginal and/or Torres Strait Islander?

- Aboriginal
- Torres Strait Islander
- Aboriginal and Torres Strait Islander
- Neither
- Prefer not to say

What is the highest level of education that you have completed

- High school - year 10 or below
- High school - year 11 or 12
- TAFE - certificate or diploma
- University - Bachelor's degree
- University - Masters degree or higher
- I have not completed any schooling

Including yourself, how many people live in your household? Please count adults and children

**About your work**

Which choice best describes the type of work that you do at the site in the location?

- Construction-focused role
- Administration-focused role
- Managerial-focused role
- Other (please specify)

Which project component do you work on (e.g. demolition, bulk earthworks, track formation etc) (please specify)

How long have you worked on this job?

- Approximately how many weeks?
- Approximately how many shifts per week?

Which shift do you usually work?

- Day
- Night
- Both

**Past experience of RAT**

Since the pandemic started, PCR based testing has been the most common form of testing for COVID-19. PCR based testing is where an individual attends a specific testing site, like a health clinic or pop-up site, and has a nasal and mouth swab performed by a health professional.

Rapid antigen tests are an alternative method for detecting the presence of COVID-19. Rapid antigen tests can be performed by the individual themselves by placing the pen, like the one pictured below, into their mouth and then into the test cartridge. This test aims to detect proteins on the surface of the COVID-19 virus from saliva in the mouth.

 Have you done a COVID-19 rapid antigen test at the site in the location?

- No
- Yes

Can you estimate the number of Rapid Antigen Tests that you have done?

- Only one test
- Between 2- 5 tests
- Between 6-10 tests
- Between 11-20 tests
- More than 20 tests

**Perceptions about rapid antigen testing at the workplace**

Please indicate your level of agreement with the following statements

|  | Strongly agree | Somewhat agree | Neither agree nor disagree | Somewhat disagree | Strongly disagree |
| --- | --- | --- | --- | --- | --- |
| I feel safer coming to work because of rapid antigen testing |  |  |  |  |  |
| It is a good thing that the rapid antigen testing is compulsory |  |  |  |  |  |
| Most of my work peers believe that rapid antigen testing is a good initiative |  |  |  |  |  |
| Most people I know on the worksite comply with the rapid antigen testing instructions |  |  |  |  |  |
| It is annoying to do the rapid antigen testing 3 times a week |  |  |  |  |  |
| I prefer to work on sites that have mandatory COVID-19 testing |  |  |  |  |  |
| I would prefer not to have to do the testing |  |  |  |  |  |
| I am concerned about the accuracy of rapid antigen tests |  |  |  |  |  |
| It is important that union representatives support the use of rapid antigen testing |  |  |  |  |  |
| My employer has provided enough information for me to understand why I have to have rapid antigen testing as often as required |  |  |  |  |  |
| The people that I live with think rapid antigen testing reduces my risk of getting COVID-19 |  |  |  |  |  |
| Rapid antigen testing is easy to do |  |  |  |  |  |
| The rapid antigen testing process does not take too much time |  |  |  |  |  |
| I would prefer to do rapid antigen testing at home |  |  |  |  |  |
| I would trust my workmates to do rapid antigen testing at home |  |  |  |  |  |

**Testing procedure**

Thinking about the last time that you did the Rapid Antigen Test, where did you do the test?

- In my car
- Outside in the car park
- At the worksite entry
- At home
- Other (please specify) ________________________________________________

Thinking about the last time you did the Rapid Antigen Test, were you alone when you did the test?

- Yes
- No

*o*

Was the person(s) you were with when you did the test, someone from your worksite?

- Yes
- No

*Display*

Including yourself, how many people were present when you did the test?

Thinking about the last time that you did the Rapid Antigen Test, how long did you hold the pen in your mouth?

- Less than 30 seconds
- About a minute
- 2 minutes
- Between 3 and 5 minutes
- More than 5 minutes
- I don't know

Thinking about the last time that you did the Rapid Antigen Test, how long did you wait for the test to finish?

- Until the control line appeared
- Less than 5 minutes
- Between 5 – 10 minutes
- 15 minutes
- More than 15 minutes

Thinking about the last time that you did the rapid antigen test, did you use a timer to measure the length of time the pen is left in your mouth and/or to wait for results

- Yes
- No

At any point, were you provided with written, verbal and/or electronic instructions to help you perform the Rapid Antigen Test?
 Please tell us which instructions you received and if they were helpful or no

|  | I received this and it was helpful | I received this and it was not helpful | I did not receive this |
| --- | --- | --- | --- |
| A video |  |  |  |
| Written instructions - either online or paper-based |  |  |  |
| Verbal instructions (3) |  |  |  |
| Other (4) |  |  |  |

In the previous question, you indicated that you were provided with verbal instructions.
 Who gave you the instructions? e.g. manager, health staff, foreman, co-worker etc.

What were their instructions?

**Rapid antigen testing outcome experience**

Q51 Which of the pictures below shows a **Negative test result**?

- A
- B
- C
- D

A ***"non-negative"*** result is when two coloured bands appear on the test, as shown on the picture below. This may indicate the presence for COVID-19 but PCR based testing is required to confirm this.
 Have you received a ***“non-negative”*** results from a Rapid Antigen Test?

- No
- Yes

If you did ever have a "***non-negative***" Rapid Antigen Test result, what would be the next steps that you would take? Please select all that apply

1. I would get out of my car to tell someone that my test was non-negative
2. I would stay in the car, and call/text someone to tell them my test was non-negative
3. I would not tell anyone about my non-negative result
4. I would do another rapid test straight away
5. I would go straight to a covid testing facility for a PCR test
6. Other (please specify) ________________________________________________

*ay This Question:*

After your ***"non-negative"*** result, how did you notify health staff and/or your manager of the result?
If you have received multiple "non-negative" results, base your answer after your last experience.

- I got out of my car to tell someone that my test was non-negative (1)
- I stayed in the car, and called/texted someone to tell them my test was non-negative (2)
- I did not tell them (3)
- Other (7) ________________________________________________

After this, what was the next step?

- I did another saliva test straight away
- I went straight to a COVID testing facility for a PCR test
- I was given access to the site
- Other (please specify) ________________________________________________

What was the outcome of the PCR test?

- Negative result--I did not have COVID
- Positive result--I did have COVID
- Unsure or I cannot remember

An ***“invalid”*** result is when the control band does not appear, as shown in the picture below.


Have you received an ***“invalid”*** result?

- No
- Yes

After your ***"invalid"*** result, how did you notify health staff and/or your manager of the result?

If you have received multiple ***"invalid"*** results, base your answer off your last experience.

- I got out of my car to tell someone that my test was invalid
- I stayed in the car, and called/texted someone to tell them my test was invalid
- I did not tell them
- Other (please specify) ________________________________________________

After this, what was your next step?

- I did another saliva test straight away
- I went straight to a COVID testing facility for a PCR test
- I was given access to the site
- Other (please specify) ________________________________________________

What was the outcome of the PCR test?

- Negative result--I did not have COVID
- Positive result--I tested positive for COVID
- Unsure or cannot remember

Have you found anything challenging about the rapid testing procedure? Select all that apply

- Remembering to pick the test up the day before my shift
- Holding the pen in my mouth for the recommended timeframe
- Waiting 15 minutes for the results of the test
- Taking the saliva sample
- The extra time added to my shift to complete the sample
- Getting a "non-negative" result and/or the process to obtain a second PCR sample
- Other reason(s) (please specify ) ___________________________________________
- ⊗I have not experienced any challenges

**Observation checklist**

| Date |  |
| --- | --- |
| Time |  |
| Site name |  |
| Person observing |  |

Test flow

| How many health staff are present? |  |
| --- | --- |
| How many people tested in this timeframe? |  |
| Are there any bottlenecks/clusters of people? |  |
| Is social distancing maintained at all times? |  |

Overall compliance of site

| Is a registered health worker overseeing the site? |  |
| --- | --- |
| Are all staff requiring recommended PPE (including gloves, mask, gown, eye protection) |  |
| What measures for infection control are in place? |  |
| Do any people undergoing testing get out of their cars before they return a negative test? |  |
| Are there any non-negatives? |  |
| Is there any evidence of non-negatives being tested again? |  |
| Is the correct procedure followed? Please record any variations to the procedure. |  |
| How are the test cartridges disposed? |  |
| Were there any concerns that privacy of individuals might be breached? |  |
| Are spare tests/stock stored in a cool dry place between 2-30°C? |  |

**Compliance of individuals**

*Observe at least 3 people doing the test*

|  | Test 1 | Test 2 | Test 3 |
| --- | --- | --- | --- |
| Is the packet opened right before the test is done? |  |  |  |
| Is the sample pen held in the mouth for two minutes (time this)? |  |  |  |
| Is the pen applied to the test pad correctly? |  |  |  |
| Does the individual wait for 15 minutes before reading the result (time this)? |  |  |  |
| Does the person use a timer to ensure the correct times are followed? |  |  |  |
| Is the correct procedure followed after the test is done? |  |  |  |
| Does the person get out of their car before returning a negative result? |  |  |  |

Overall comments:
